# Supplementary material for: Acceptability of risk-based triage in cervical cancer screening: A focus group study
Source: PLoS One. 2023 Aug 16;18(8):e0289647. doi: 10.1371/journal.pone.0289647 (PMC10431661; doi:10.1371/journal.pone.0289647)
Supplement: S2 Table — (DOCX) [file pone.0289647.s002.docx]

S2 Table. *Topic guide*

| **Topic** | **Questions** |
| --- | --- |
| **Risk-based triage in cervical cancer screening: introduction** | - Short introduction round (name, why participate in the study?) - What is your first reaction to risk-based triage in cervical cancer screening?   o What could be the benefits of risk-based triage?  o What could be the disadvantages of risk-based triage?   - What is your reaction to the following scenarios:   o Tanja is 50 years old. She tested positive for HPV and no abnormal cells were found. Based on other factors, Tanja has little chance of developing cervical cancer. Tanja is therefore invited for a check-up smear after 12 months, instead of after 6 months.  o Esther is 40 years old. She tested positive for HPV and no abnormal cells were found. Esther has an increased risk of cervical cancer based on other factors. Esther is therefore invited again after 3 years to participate in cervical cancer screening, instead of after 5 years.  o Naima is 30 years old. She also tested positive for HPV and no abnormal cells were found. Based on the other factors, Naima has very little risk of cervical cancer. Naima therefore is not invited for a check-up smear. |
| **Cervical cancer risk factors** | - There are a number of factors that can influence the risk of cervical cancer. - Per risk factor:   o What is your opinion on being asked about this in a questionnaire?  o The risk factors:   - Age - Education Level - Marital status - Parity / age at first birth - Type of contraception - Smoking - Use of immunosuppressants - Other HPV infections (e.g. genital warts) - STDs - Number of lifetime sexual partners - Age at first sexual contact - There are different types of HPV. Some types of HPV increase the risk of cervical cancer more than other types of HPV. What do you think about on including HPV type when deciding whether to refer someone? |
| **Information needs and stigma** | - Suppose risk-based triage is introduced and you are invited to participate in cervical cancer screening, what would you like to know? - Would you like to know your own risk of cervical cancer?   o How would you prefer to learn your own risk?  o Would you like to know any additional information?   - What would be your reaction if you were told that you have a low risk of cervical cancer? - What would be your reaction if you were told that you have a high risk of cervical cancer? - While organizing these conversations, I was in contact with a woman who was interested but ultimately refused to participate because she had once received a pap result. She was worried that the rest of the group would judge her negatively, because people would link these results to sexual behavior. What is your view on this? Do you think this can be a problem with risk-based triage, that people link a higher risk score to sexual behavior? |
| **Risk-based triage in cervical cancer screening: conclusion** | - Now you've heard a bit more about risk-based triage and the cervical cancer risk factors, what do you think about this now? - What would you think when a friend is invited for a more intensive screening program because she is at greater risk? - Could risk-based triage make that you do or do not want to participate in cervical cancer screening? - To what extent do you discuss cervical cancer screening with friends and family? o Would this change if the program changed to a more risk-based program? |
| **Closing remarks** | - Anything else you would like to pass on to us? - Thank you for your participation. |
