# Supplementary material for: Acceptability of risk-based triage in cervical cancer screening: A focus group study
Source: PLoS One. 2023 Aug 16;18(8):e0289647. doi: 10.1371/journal.pone.0289647 (PMC10431661; doi:10.1371/journal.pone.0289647)
Supplement: S3 Table — (DOCX) [file pone.0289647.s003.docx]

S3 Table. *Demographic and cervical cancer screening characteristics per participant*

| **FGD** | **Participant** | **Age** | **Education level** | **Ever participated cervical cancer screening?** | **Ever tested positive for HPV?** | **Ever had a check-up smear or a referral to the gynecologist?** |
| --- | --- | --- | --- | --- | --- | --- |
| 1 | 1 | 49 | higher | yes | no | yes |
|  | 2 | 56 | lower-intermediate | yes | no | yes |
|  | 3 | 58 | lower-intermediate | yes | no | yes |
| 2 | 4 | 36 | higher | yes | yes | yes |
|  | 5 | 54 | higher | no | NA | NA |
| 3 | 6 | 51 | higher | yes | no | no |
|  | 7 | 30 | higher | no | NA | NA |
|  | 8 | 46 | higher | yes | no | no |
|  | 9 | 50 | lower-intermediate | no | NA | NA |
| 4 | 10 | 49 | higher | yes | no | no |
|  | 11 | 47 | lower-intermediate | yes | no | yes |
|  | 12 | 36 | lower-intermediate | yes | yes | yes |
|  | 13 | 46 | higher | yes | no | no |
|  | 14 | 54 | lower-intermediate | yes | no | yes |
|  | 15 | 44 | lower-intermediate | yes | yes | no |
|  | 16 | 53 | lower-intermediate | yes | don’t know | yes |
| 5 | 17 | 42 | lower-intermediate | no | NA | NA |
|  | 18 | 45 | higher | yes | yes | no |
|  | 19 | 35 | lower-intermediate | yes | yes | yes |
|  | 20 | 46 | lower-intermediate | yes | no | no |
|  | 21 | 46 | higher | no | NA | NA |
| 6 | 22 | 35 | higher | yes | yes | no |
|  | 23 | 35 | higher | no | NA | NA |
|  | 24 | 36 | lower-intermediate | yes | don’t know | yes |
|  | 25 | 37 | higher | no | NA | NA |
|  | 26 | 48 | lower-intermediate | yes | don’t know | no |
| 7 | 27 | 53 | lower-intermediate | yes | no | no |
|  | 28 | 48 | higher | yes | no | no |
