# Supplementary material for: Acceptability of risk-based triage in cervical cancer screening: A focus group study
Source: PLoS One. 2023 Aug 16;18(8):e0289647. doi: 10.1371/journal.pone.0289647 (PMC10431661; doi:10.1371/journal.pone.0289647)
Supplement: S4 Table — (DOCX) [file pone.0289647.s004.docx]

S4 Table. *Example quotes per (sub)theme*

| **Theme** | **Subtheme** | **Quotes** |
| --- | --- | --- |
| Adequate screening program | Evidence-based | “I would especially want to be assured that there are no greater risks because people are tested less often. So I would also like to see a little more evidence for changing this guideline.” (FGD3) |
|  |  | “Being married, doesn’t say anything about your sexual behavior, of course.” (FGD4) |
|  | (Pre-)cancer detection | “That for me as a person the chance that I do have it and that it will not be picked up does not increase. That would be relevant to me.” (FGD3) |
|  |  | "I think it's good that they're on top of it, which reduces the risk of you actually getting seriously ill and dying." (FGD2) |
|  | Accurate risk classification | "Who's to say she neatly sticks with her own partner? And not get a divorce next year, which might put her at high risk again?" (FGD4) |
|  |  | " But I also think that it depends on who you have in front of you, whether you get an honest answer to that." (FGD2) |
|  | Trust | “If I know that there will be a well-thought-out questionnaire and certain steps are taken, then I feel reassured enough.” (FGD5) |
|  |  | “Yes, I think it just feels too odd or too scary, because you're so used to current practice. The HPV [screening] is actually also recent, but you really trust how things are going now.” (FGD3) |
|  | Less unnecessary testing | “I think that's a very good thing that unnecessary examinations are not being done.” (FGD6) |
|  |  | “Because of course it is also very nice for the patient herself that they do not have to go through a whole medical process that may not lead to anything at all.” (FGD5) |
| Personal information | Intrusiveness | “I don't think it will be a fun questionnaire if you have to give a lot of positive answers.” (FGD3) |
|  |  | “I think that some people withhold things or don't fill them in and maybe a can of worms will be opened that should stay closed.” (FGD4) |
|  | Stigma / no fear of stigma | “If a friend were to say that to me, my first reaction would just be: ‘how awful’. But I wouldn't think about it like: ‘why is she at higher risk?’ No, I don’t think so." (FGD3) |
|  |  | “In principle, no one else will know. Unless you tell them.” (FGD2) |
|  | Understanding relevance | “Why do I have to answer this? What's in it for me? And when you know that, I think a lot of barriers are removed.” (FGD4) |
|  |  | “In any case, it should be possible to find it. Because I can really imagine: it's about my cervix, it's not about my education. I can imagine that people would like to be able to read that information somewhere.” (FGD3) |
|  | Privacy | “But I think you should include the option that they don't have to fill it in. Because otherwise you might get a wrong [answer]. I would find it much worse if someone fills in that it is all 'no', out of embarrassment or maybe because they’re also a little scared: ‘what happens to that data, will other people find out?’.” (FGD3) |
|  |  | “The more difficult or the more intimate the question, the more important security is.” (FGD7) |
| Emotional impact | Fear of having cancer | “I sometimes have to wait for check-up results and that just causes a lot of stress. And knowing that you have a low risk is more pleasant than thinking: ‘I have to come back, because I have a very high risk’, while it turns out not to be the case.” (FGD1) |
|  |  | “Then I immediately get to the scary thing of risk-based: that you do not belong to a large group, who are all invited, so you can easily think: ‘this is just standard, nothing to worry about, I just have to do this’. While if it is personalized, you will naturally wonder, 'hey, why me and not the neighbor?'” (FGD1) |
|  | Reassurance | “What makes you are invited for follow-up, and the other person is not? Yes, you should know that. I mean, because otherwise people will start googling and stuff and they also see that the neighbor is not invited and you are. Then you're just going to make things up yourself, that's even worse I think. I think you would come to the same thing as with the Pap. That that also comes with an explanation of 'if your category is this and category such and such, then it is not a very high risk, but we will do it just to be sure'.“ (FGD1) |
|  |  | “[I think] it can also be reassuring that you can fill in that [the questionnaire], because then you know that you are being monitored closely.” (FGD6) |
|  | Fear of missing cancer | “The question is: do you feel fooled if you do eventually get sick?” (FGD2) |
|  |  | “If I know that I have even a small risk of something and it is never looked at again... I personally would find that complicated for myself.” (FGD1) |
| Communication | Transparency | “That you simply have no guarantee that everything will be found. It is of course the intent that you catch as much as possible and do as little harm as possible with the tests. But that you will never be able to guarantee that you will catch everything.” (FGD3) |
|  |  | “It reminds me of the dentist. If I got a check-up and I didn't have cavities for a few years in a row, then he said: '[…] you can come once a year. And if you have a cavity, you have to come every six months.’ The reason behind it is explained. If you know why, it is already a lot clearer.” (FGD4) |
|  | Personal contact | “I like an in-person meeting better, because a letter is so impersonal. If I get questions during such a conversation, I can immediately ask them, because then, I think, I will also be reassured.” (FGD5) |
|  |  | “But then I would like to talk to the GP about it, like: hey, what are the risks, and what is the policy then, and more things. Every person has different personal questions. You have a set of general questions, of course, but also personal questions, which I don't think can ever be answered in a letter." (FGD7) |
|  | Accessibility | “I think that a combination with pictures - you can make it a bit visual - makes it more accessible for many people. So that you also understand it better than when you have to read a whole piece of text, with numbers. You quickly read over that, and think: yes, sure, yes.” (FGD6) |
|  |  | “You probably have to describe it very graphically if you want to get a clear answer.” (FGD2) |
| Autonomy | Education and prevention | “Knowledge is nice, knowing which risk factors have made you receive that invitation, so that you can also take this into account in the future and act accordingly. Changing your lifestyle.” (FGD6) |
|  |  | “In the first, second year of high school with biology lessons, they also get the part of safe sex and condom use. ‘These are STDs that you can get like that.’ But I think that should include HPV as well.” (FGD5) |
|  | Freedom of choice | "Suppose someone is just a more anxious woman, for that woman it might be nice to be referred anyway to rule it out." (FGD7) |
|  |  | “I don't need to know my risk of everything all the time, so I'd really want to think about that: how much do I want to know?” (FGD6) |
