# Supplementary material for: Acceptability of risk-based triage in cervical cancer screening: A focus group study
Source: PLoS One. 2023 Aug 16;18(8):e0289647. doi: 10.1371/journal.pone.0289647 (PMC10431661; doi:10.1371/journal.pone.0289647)
Supplement: Attachment — Submitted filename: Review comments.pdf [file pone.0289647.s005.pdf]

Review comments: PONE-D-23-06691

Dear Editor,

Thank you for giving me the opportunity to provide a peer review for this research article titled, "Acceptability of risk-based triage in cervical cancer screening: a focus group study". The article explored the factors that influenced the acceptability of risk-based triage cervical cancer screening among women 30-60 years in The Netherlands.

While I consider this paper valuable because it provides useful findings on the topic and should be accepted for publication after the following improvements have been made to the article. Find below my comments that could improve this work.

1. Could the authors describe how they selected the four general practices for the study?
2. Could you state the number of participants recruited for the study in the method section?
3. In line 33 age group should be moved from the end of the sentence. The sentence should read "Therefore, it is essential to gain knowledge of the views of women (30-60 years) eligible for cervical cancer screening in The Netherlands".
4. In lines 51 and 52 could you highlight any one of the challenges with risk-based triage that needs attention?
5. Years should be added after women in line 62.
6. Lines 67 to 70 should read "As a consequence, the detection rate for 68 cervical intraepithelial neoplasia grade 2 or worse (CIN2+) increased from 999 to 1461 per 69 100,000 among women screened between 2014/2015 and 2017/2018. On the other hand, clinically irrelevant findings (≤CIN1) increased from 611 to 1487 per 100,000 among women screened".
7. I am not clear about how the sentence in lines 84 and 85 relates to the research topic, especially so because apart from this sentence nowhere in the paper have you suggested the use of machine learning in this intervention. Could you provide further clarification?
8. In line 116 I suggest you re-write the sentence "An information e-mail, which provided brief study information and the researchers' contact details, was sent to all female patients aged 30 to 60 years old who were registered in four general practices" to read "An email which provided brief information about the study and the researchers' contact details, were sent to all female patients aged 30 to 60 years old who were registered in four general practices".
9. Provide the other dichotomized category for educational level in line 130.
10. In lines 155 and 156 the authors reported that in "the largest FGD, engagement varied because of several reasons, such as distractions at home". How did these distraction impact effective participation and responses from participants?
11. In line 357 the authors indicated that "one of the strengths of the study was the diverse sample with regard to age, education level, and cervical cancer screening experience". However, the study excluded non-Dutch-speaking women. How has this exclusion impacted the results of this study?
