# Supplementary material for: Acceptability of risk-based triage in cervical cancer screening: A focus group study
Source: PLoS One. 2023 Aug 16;18(8):e0289647. doi: 10.1371/journal.pone.0289647 (PMC10431661; doi:10.1371/journal.pone.0289647)
Supplement: Attachment — Submitted filename: PLOS One review.docx [file pone.0289647.s006.docx]

PLOS One review

This paper should be published after a few edits and clarifications. The authors need to review the manuscript heavily for grammar.

General comments: The paper must be reviewed for grammar and spelling errors, i.e. see line 73 “…women is, since July 2022, based on..”

Methods:

Study design:

Lines 103: The author should provide more details on the design. Stating that it is a qualitative focus group does not tell readers what the methodology entails.

Line 103-108: Informed consent and ethical approval should move to the ethics section.

Line 133: If your preferred age range is 30 and 60 years old women, the statement about maximizing heterogeneity of women participating may be faulty. The authors already have a set parameter that limits true heterogeneity based on age. I suggest removing age and focusing on educational level, socioeconomic status, and CCS experience.

Line 136: Interview guide: I understand the need to call it an interview guide, but in a focus group discussion, while a group interview, the interviewer is more a facilitator than an interviewer. This leads back to my comment clarifying exactly what a qualitative focus group study means.

Line 154: “…on average four participants per FGD (2-7 participants)…” FDGs should be a minimum of 3 participants.

Lines 160-162: “…and were transcribed verbatim. Information that could lead to identification of participants, such as name or place of residence, was not included in the transcripts. The transcripts were not returned to the participants.” This should go down to analysis.

Line 170: citation

Line 177: (re)named. Why is this in parenthesis?

Results:

Line 244: (*FGD3, p8, aged 46)* Unless there is a specific reason why you need this level of detail, remove the page number.
