# Supplementary material for: Acceptability of risk-based triage in cervical cancer screening: A focus group study
Source: PLoS One. 2023 Aug 16;18(8):e0289647. doi: 10.1371/journal.pone.0289647 (PMC10431661; doi:10.1371/journal.pone.0289647)
Supplement: Attachment — Submitted filename: Response feedback reviewer 2 - authors response.docx [file pone.0289647.s007.docx]

Review comments: PONE-D-23-06691

Dear Editor, Thank you for giving me the opportunity to provide a peer review for this research article titled, “Acceptability of risk-based triage in cervical cancer screening: a focus group study”. The article explored the factors that influenced the acceptability of risk-based triage cervical cancer screening among women 30-60 years in The Netherlands.

While I consider this paper valuable because it provides useful findings on the topic and should be accepted for publication after the following improvements have been made to the article. Find below my comments that could improve this work.

1. Could the authors describe how they selected the four general practices for the study?
**Author’s response**: We thank the reviewer for this helpful suggestion. We added information about the selection of the four general practices (line 117-120): “The general practices were selected because they are core practices in the Radboudumc General Practice Network and are familiar with participating in scientific research. The practices are located in different neighborhoods in the city of Nijmegen and cover a broad range of socioeconomic status and ethnic backgrounds.”.

2. Could you state the number of participants recruited for the study in the method section?
**Author’s response**: Indeed, the number of participants should have been included in the methods section. Therefore, we changed the sentence (line 156): “In total, seven FGDs were held with on average four participants per FGD (2-7 participants).” into “Seven FGDs were held with 28 participants in total (2-7 participants per FGD).”.

3. In line 33 age group should be moved from the end of the sentence. The sentence should read “Therefore, it is essential to gain knowledge of the views of women (30-60 years) eligible for cervical cancer screening in The Netherlands”.
**Author’s response**: Agree. The age group has been moved as suggested by the reviewer (line 32).

4. In lines 51 and 52 could you highlight any one of the challenges with risk-based triage that needs attention?
**Author’s response**: We agree that this information would be valuable to the abstract. Therefore, we have decided to shorten the abstract in order to add (line 52-53): “These challenges include dealing with sensitive topics and a transparent communication strategy.”

5. Years should be added after women in line 62.
**Author’s response**: We have changed this line into “[…] for all women aged 30-60 years” (line 61).

6. Lines 67 to 70 should read “As a consequence, the detection rate for 68 cervical intraepithelial neoplasia grade 2 or worse (CIN2+) increased from 999 to 1461 per 69 100,000 among women screened between 2014/2015 and 2017/2018. On the other hand, clinically irrelevant findings (≤CIN1) increased from 611 to 1487 per 100,000 among women screened”.
**Author’s response**: We have incorporated this suggestion (line 66-70).

7. I am not clear about how the sentence in lines 84 and 85 relates to the research topic, especially so because apart from this sentence nowhere in the paper have you suggested the use of machine learning in this intervention. Could you provide further clarification?
**Author’s response**: We thank the reviewer for pointing this out. The sentence about machine learning was included to give an example of our statement that the population benefit-harm ratio of cervical cancer screening can be improved by an optimized risk-based scenario. However, we agree that this sentence does not fit perfectly in our introduction. We have now removed the part about machine learning to emphasize the results rather than the methods (line 83-84): “A recent study has indeed shown that accurate risk classification can be improved to develop a personalized cervical cancer screening program (19).”

8. In line 116 I suggest you re-write the sentence “An information e-mail, which provided brief study information and the researchers’ contact details, was sent to all female patients aged 30 to 60 years old who were registered in four general practices” to read “An email which provided brief information about the study and the researchers’ contact details, were sent to all female patients aged 30 to 60 years old who were registered in four general practices”.
**Author’s response** : We thank the reviewer for these helpful suggestions. We have revised these lines (line 115-117).

9. Provide the other dichotomized category for educational level in line 130.
**Author’s response**: We agree that both education level categories should be further explained. Therefore, we have changed the part about educational level to add more information about the lower-intermediate category. The original sentence was “Education level was dichotomized, and a higher education level was defined as having completed university or university of applied sciences, which is consistent with the classification of Statistics Netherlands (22).”. The new sentences read as follows (line 130-134): “Education level was dichotomized consistent with the classification of Statistics Netherlands (22). A higher education level was defined as having completed university or university of applied sciences, and a lower-intermediate education level includes primary education, prevocational secondary education, senior general secondary education, pre-university education, and senior secondary vocational education.”

10. In lines 155 and 156 the authors reported that in “the largest FGD, engagement varied because of several reasons, such as distractions at home”. How did these distraction impact effective participation and responses from participants?
**Author’s response**: We understand that this sentence raises the question whether/how the variable engagement impacted the responses from the participants. Therefore, we have added the following (line 159-160): “However, the (partial) absence of some participants did not negatively impact the active discussion between the other participants.”.

11. In line 357 the authors indicated that “one of the strengths of the study was the diverse sample with regard to age, education level, and cervical cancer screening experience”. However, the study excluded non-Dutch-speaking women. How has this exclusion impacted the results of this study?
**Author’s response**: We decided to only include Dutch-speaking women in order to conduct the FGDs in Dutch. English-speaking FGDs would exclude women who are not proficient in English, and we did not aim to conduct an FGD with only non-Dutch-speaking women. We agree with the reviewer that a diverse sample is important, and we do agree that we might have missed some perspectives by excluding this group. We hope future research will study the perspectives of a broader group of women, and we added non-Dutch-speaking women to our paragraph about future research (line 375-377): “In future studies, more attention is needed on the perceptions and views of non-Dutch-speaking women and of women who are harder to reach, including women who do not intend to participate in the current cervical cancer screening program.”
